# Beta-band oscillations accompanying fast, targeted ankle plantarflexions in older adults: the effects of resistance training

**DOI:** 10.1016/j.nbas.2026.100160

**Published:** 2026-07-11

**Authors:** S. Walker, S. Sipilä, H. Pesonen, I.M. Tarkka, J.P. Ahtiainen, T. Parviainen, J. Kujala

**Affiliations:** a NeuroMuscular Research Center, Faculty of Sport and Health Sciences, University of Jyväskylä, Finland; b Gerontology Research Center, Faculty of Sport and Health Sciences, University of Jyväskylä, Finland; c Centre for Interdisciplinary Brain Research, Department of Psychology, University of Jyväskylä, Finland

**Keywords:** Sensorimotor, Aging, Cortical oscillations, Strength, Motor beta rhythm

## Abstract

This study evaluated short- and long-term resistance training on cortical movement-related beta-band oscillations, purportedly an indicator of somatosensory processing, in older adults. A cross-sectional comparison between long-term resistance-trained (*n* = 10) versus untrained individuals (*n* = 8) and a longitudinal 12-month intervention of short-term resistance training (*n* = 20) were employed in >70-year-old adults. Cortical activity was recorded by 306-channel whole-head magnetoencephalography (MEG) inside a magnetically shielded room. Participants underwent maximal unilateral plantarflexion force tests using a custom-built MEG-compatible dynamometer, from which 15% and 50% force levels were displayed on a screen. Thereafter, participants performed isometric contractions as fast and as accurately as possible to the target force line and relaxed as fast as possible. Thirty trials to each force level (15% and 50%) were performed sequentially. Average force, force and timing coefficient of variation, and rate of force development were analysed from both force levels. Only 15% trials were viable for MEG analysis, examining peak beta (10–30 Hz) suppression and rebound frequency, amplitude and timing, due to large magnitude artifacts from 50% trials. Long-term training was accompanied by lower relaxation time coefficient of variation (*P* = 0.021–0.027). After short-term training, beta rebound amplitude increased (∼107Δ%, *P* = 0.003) and time to peak rebound reduced (∼1.49 to ∼1.27 s, *P* = 0.039) with similar but non-significant differences between long-term trained and untrained individuals (∼1.27 vs. ∼1.57 s, *P* = 0.083). Both short- and long-term effects suggest more efficient sensorimotor processing and lower force variability accompanying resistance training in older age.

## 1 Introduction

Muscular contraction and movement are characterized by alterations in somatosensory activity leading to an efferent command and subsequent afferent feedback [1]. Accurate force regulation, which is reliant on the efficacy of such neural activity, is essential to perform the desired motor action. One area of scientific interest regarding motor control is beta-band oscillations (∼15–30 Hz) that accompany passive [2], [3] and voluntary movement [4], [5]. Prior to muscular contraction beta-band power is transiently reduced, typically lasting the duration of the contraction [5], which has been termed event-related desynchronization or beta suppression. Thereafter, a brief increase in beta-band power above baseline is observed [6], which is termed event-related synchronization or beta rebound. The primary location of beta suppression appears to be the primary somatosensory cortex (SM1) [2] while the origin of beta rebound appears to be the primary motor cortex (M1) [7]. Gamma-aminobutyric acid (GABA)_A_ activity has been shown to play a role in beta suppression, with GABA_B_ activity modifying the beta rebound [8].

Older age has been shown to accompany greater beta suppression amplitude [3], [4], [9] during a variety of button press and passive movement experiments. This greater, baseline normalized, beta suppression may be due to a consequence of (a need to overcome) higher baseline beta-band power [3], [10], and it has been speculated to be a consequence of impaired somatosensation. Further, neurodegeneration through amyotrophic lateral sclerosis and its gene mutation has been associated with greater beta suppression (Proudfoot et al. 2017). Regarding beta rebound, studies have reported mixed results with some showing greater rebound [9] and some weaker rebound [4] amplitude in older age, and some showing no difference [3]. The timing of the beta rebound may be more consistent than its amplitude in distinguishing between age-groups, being delayed in older age [3], [4], [9], although differences in task cannot be discounted as a possible confounding factor. Nevertheless, convincing evidence shows that neurological disease reduces the amplitude of the beta rebound [11], [12], [13], which accompanies reduced force accuracy [11] and hand dexterity [12] while a relatively stronger rebound is associated with better post-stroke recovery [13]. Thus, beta oscillations during and following muscular contraction could indicate cortical somatosensory processing and its possible decline during aging.

Previously, older adults have demonstrated clear deficiencies in maximum force production, maximum rate of force production, and submaximal force steadiness compared to younger adults [14]. Further, follow-up studies have shown a rate of decline of approximately 2% annually in maximum force production (e.g. [15]). The lower maximal force values are accompanied by reduced motor unit discharge patterns and voluntary activation level, while the poorer force steadiness performance is accompanied by higher variability in motor unit discharge rates [14]. Overall, aging seems to be characterized by increased variability in neural control of force production tasks, although healthy older adults retain the capacity to adapt motor unit functioning through resistance training in a similar manner as young adults [16].

A key limitation in aging research is that humans tend to reduce their amount of physical activity as they age [17]. This reduction in muscular contraction and movement *‘practice’* may contribute to alterations in beta-band oscillations and complicate interpretations on aging effects. It has been suggested that a better model to assess the effects of aging is to utilize highly trained individuals, who maintain high levels of physical activity throughout life [18]. Based on this rationale, two complimentary experiments were used to determine the effects of resistance training on beta-band oscillations to rapid muscular contraction. First, a cross-sectional comparison between long-term (10 years) resistance trained and untrained counterparts (the SARCOPENIA study) and, second, a (12 month) longitudinal resistance training intervention in previously untrained older adults (the PASSWORD study). Together, these experiments provide an opportunity to clarify the knowledge gap of whether movement-related beta-band oscillations are trainable and have potential relevance to aging motor control.

The aim of this study is to determine the effects of short- and long-term resistance training on beta-band responses to voluntary lower-limb contractions. We hypothesized that: 1) faster rate of force development (RFD) and more accurate force production and 2) reduced cortical beta-band suppression and stronger rebound would occur in the trained state.

## 2 Materials and methods

### 2.1 Study design and data source

To examine the effects of long-term (ten years) and short-term (one year) resistance training on force production and beta-band oscillations. The data was derived from two studies, the SARCOPENIA study [19] and the PASSWORD study ([20]; ISRCTN52388040). Both studies were provided with statements from ethical review boards (SARCOPENIA = The Ethical Committee of the University of Jyväskylä, ref.: 4/2017; PASSWORD = The Ethical Committee of Central Finland Health care District, ref.: 11/2016).

The SARCOPENIA study involved a supervised resistance training intervention for 12 months in 2007 followed by 10-year follow-up, from which 19/35 participants continued with regular non-supervised resistance training approximately twice per week [21]. Based on annual questionnaires (i.e. recall), participants reported using resistance machines (e.g. leg press, knee extension and flexion, pull-down, shoulder press) predominantly, although some free-weight exercises, such as back-squat, bench press and biceps curl were also reported. Most training followed a whole-body programme with either body-weight or machine-based abdominal (e.g. crunches) and lower back exercises (e.g. Roman chair back extension). Approximately 6–10 exercises were performed in each training session and the typical set and repetition ranges for the exercises were 2–4 and 6–12, respectively.

The PASSWORD study was a randomized controlled trial where participants were allocated to multicomponent physical exercise training including resistance training or multicomponent physical exercise + cognitive training several times per week. A sub-group of volunteers (*n* = 28, equal numbers per group) agreed to additional MEG measurements, and were measured at 0, 6 and 12 months of the intervention. The 12-month intervention for all participants consisted of two supervised training sessions per week; with one session focussed on walking and balance exercises while the other used pneumatic resistance machines and SmartCard technology to implement progressive resistance training [20], [22]. In total, 8–9 resistance exercises were performed for whole-body training, beginning with 2 sets and 15–20 repetitions per exercise progressing to 3 sets of 6–10 repetitions, and a final emphasis being on high-velocity intention. In addition, unsupervised, home-based resistance training was performed 2–3 times per week focussed on the hip and knee extensors, with the initial resistance being body-weight (0–6 months) that progressed to use of resistance bands (7–12 months).

Cognitive performance and accompanying cortical data from the Stroop test [22] and cortical P3 responses to electrical stimulation [21] have already been published. Thus, the present data is unique and not previously reported.

### 2.2 Participants

Volunteers from the SARCOPENIA study were 10 resistance-trained males (age = 78 ± 2 years, height = 175 ± 5 cm, body mass = 82 ± 11 kg), who had engaged in regular gym-based training for the past 10 years, and 8 sex- and age-matched controls (age = 78 ± 3 years, height = 173 ± 8 cm, body mass = 78 ± 9 kg), who were physically active and engaged in low-intensity daily activities (e.g. walking, cycling, gardening etc.). Volunteers from the PASSWORD study were community-dwelling sedentary or at most moderately physically active older males and females who had no resistance training history. Twelve of them were assigned to the physical exercise training group (7 females, age = 76 ± 3 years) and eight to the physical exercise + cognitive training group (8 females, age = 73 ± 3 years). For the purposes of the present study, the two groups were collapsed since both engaged in regular resistance training 2–3 times per week over a 12-month period (see [22] for intervention details). Only participants whose MEG data were of good quality and not compromised by excessive movement artifacts were included in the present study (i.e. 5 removed due to artifacts from movement during contractions). As this was an exploratory study, incorporating a unique sample of long-term resistance-trained older adults, it was not possible to perform sample size estimations. Nevertheless, the number of participants in each of the groups is typical for such studies.

### 2.3 MEG measurements

Measurements for SARCOPENIA participants were performed in 2017 and PASSWORD participants in 2017–2018. The SARCOPENIA participants visited the lab once for measurements. The PASSWORD participants performed baseline measurements (Pre-training) and repeat measurements after 6 (Mid-training) and 12 (Post-training) months of physical exercise training. All measurements involved the same unilateral isometric plantarflexion task and identical analysis procedures.

Participants were firmly secured to a custom-built force dynamometer (University of Jyväskylä, Finland), where a commercial MEG chair (Elekta Oy, Helsinki, Finland) was modified and non-magnetic strain gauges inbuilt to aluminium plates fixed to the leg supports of the chair. The footplate was positioned under the participant's 1st metatarsal-phalanx joint of the dominant limb (participants answered a question as to which foot they prefer to kick a ball with). The foot, shin and thigh were secured to the chair with non-elastic straps to prevent movement.

First, the participants performed a brief warm-up consisting of multiple submaximal isometric plantarflexions and then maximum voluntary contraction force (MVC) was obtained by performing 3–5 maximal contractions separated by 1 min recovery between trials. Real-time force feedback was displayed on a screen 2–3 m in front of the participant with 10 s sweeps. Once a stable MVC (i.e. within 5% of the second-best trial) was identified, target lines at 50% of MVC and 15% of MVC were displayed. Participants were instructed to plantarflex as fast and as accurately as possible to raise the force level to the target line and then immediately stop contracting, such that the force trace would rise and fall as fast as possible. Two vertical guidelines were added at 3 and 7 s so that one contraction would be performed during one sweep. This also ensured at least 6 s time-lapse between contractions. Otherwise, the participants could freely choose when to initiate the contraction. The tester was inside the magnetically shielded room (VacuumSchmelze GmbH, Hanau, Germany) with the participant to provide direct feedback and verbal encouragement during the MVC trials and the practice trials of the plantarflexion task.

#### 2.3.1 Force data recordings

For the test trials, the tester withdrew from the room, and the door was closed. Force data was recorded in Signal 4.04 software (CED, Cambridge, UK) via a 16-bit A/D converter (Micro1401–4, CED, Cambridge, UK) to a pc. The force amplifier and recording equipment were located outside of the magnetically shielded room. The participant could be contacted through an audio system that allowed the tester to provide feedback on the performance of each trial to ensure stable and correct contractions, as instructed. Thirty trials to 50% of MVC were performed followed by thirty trials to 15% of MVC separated by 3–5 min of rest. The number of trials was chosen to obtain the highest number of contractions without inducing fatigue, based on our pilot measurements (i.e. 30 trials for 50% of MVC and 60 trials for 15% of MVC).

#### 2.3.2 MEG data recordings

MEG data was collected using a 306-channel whole head Elekta Neuromag system (Elekta Oy, Helsinki, Finland) in a magnetically shielded room at the Jyväskylä Centre for Interdisciplinary Brain Research. Data were filtered at 0.03–330 Hz and sampled at 1000 Hz. The participants were seated, with the head covered by the MEG helmet. Each participant's head position with respect to the MEG sensor array was determined by attaching five head position indicator coils to the scalp and briefly energizing them before the measurement. The coil locations were determined in reference to anatomical landmarks (nasion and right/left preauricular points) using a 3-D digitizer (Isotrak 3S1002, Polhemus Navigation Science). Blinks and eye movements (saccades) during the MEG measurement were monitored using electro-oculography (EOG).

#### 2.3.3 Force data analyses

Analyses occurred offline and was performed by a customized script. The force signal was first filtered using a 4th-order Butterworth low-pass filter at 10 Hz. Thereafter, the maximum force recorded during the 50% and 15% of MVC target force trials (i.e. 100%) was identified and a 1% increase from baseline marked the contraction initiation. The slope of the force curve from its 1% initiation to the 100% force level was taken as the RFD. From the two force variables (maximum and RFD), the average of the thirty trials was calculated along with their standard deviation (SD) and coefficient of variation (CV). Additionally, an error ratio was calculated as the average force divided by the target force. Relaxation time was also identified as the time from the maximum force until the first data point when the force fell below the baseline (mean + 2SD, 200 ms time-window, −2 to −1.8 s prior to contraction). Again, SD and CV from relaxation time were calculated. Contraction time, from force initiation to end, was also obtained.

#### 2.3.4 MEG data analyses

To synchronize MEG data with force data, the dynamometer signal was sent to an auxiliary port in the Elekta Neuromag system and contraction initiation was identified as mean + 2SD of force application during the baseline time-window (−2 s to −1.8 s before contraction initiation). MEG data were pre-processed with MNE and MNE-python [23], [24]. For both studies, spatiotemporal signal space separation (tSSS) was applied to suppress external disturbances from the MEG data and to transform the MEG to a uniform head position [25]. Additionally, for the PASSWORD data, sensor-specific noise was suppressed using the oversampled temporal projection method [26] and independent component analysis was used to suppress ocular and cardiac artifacts.

**Fig. 1:**
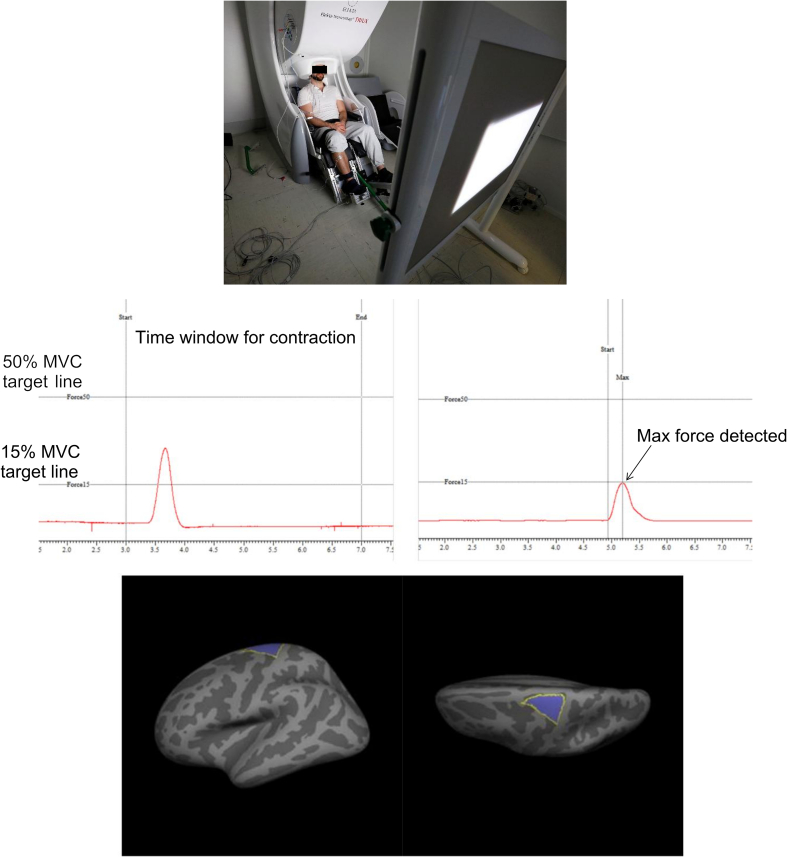
Experimental set up with the participant sat in the modified MEG chair, ankle, thigh and waist secured by non-elastic strapping and force trace displayed on the screen (top). Force traces of two participants showing the raw trace obtained during data collection of an inaccurate trial with Start-End cursors between which a contraction should be performed (middle left) and an accurate trial of filtered data with contraction initiation (Start) and peak force (Max) identified (middle right). In both cases, the target force level was 15% of MVC (“Force15”). Region of interest (ROI) anatomically representing the foot/leg motor area used in MEG data analyses (bottom).

Estimation of neural activity in the motor cortex with respect to the contraction was estimated using MNE and MNE-python. As individual-level structural magnetic resonance images were not available, these analyses were conducted in a template brain in Freesurfer [27]. Here, we focused on a single region of interest (ROI) representing the foot/leg motor area (Fig. 1). The ROI was based on a modified version of the automatic anatomic parcellation of human cortical gyri and sulci [28], where the precentral gyrus parcel was split into 5 smaller parcels (see Ala-Salomäki et al., 2021). The source-level estimation of activity was conducted using cortically constrained noise-normalized L2-minimum-norm estimation where empty-room data was used to determine the noise-covariance. Neural activity was estimated in the 42 source-points within the foot/leg motor cortex ROI. These time-series were low-pass filtered at 40 Hz. Trials for which any of the gradiometers showed amplitudes of more than 4000 fT/cm were excluded from the analyses. As the MEG data from the contractions to 50% of MVC contained prominent artifacts across participants, the MEG analyses focus only on contractions to 15% of MVC.

Modulation of beta-band activity was examined within the ROI via time-frequency representation (TFR) analyses. Here, activity in the beta band from −2 to 2 s with respect to contraction initiation was estimated at 10–30 Hz at 1 Hz steps using Morlet wavelets of width 7. Here, the data were downsampled by a factor of 10. The data were baseline-corrected using the interval of −1.5 to −1 s with respect to contraction initiation. Although specific test-retest reliability data are not available for the plantarflexion contractions used in the current experiment, several studies have demonstrated excellent reliability (i.e. ICC ≥ 0.9) for beta amplitude [29], peak beta frequency [30], and peak beta latency [31] using similar methods, and these levels of reliability are stable over 1-year of repeat testing [31].

### 2.4 Statistical analyses

Statistical analyses were performed in SPSS software (version 30, IBM ltd, Armonk, USA). Most variables did not fulfil the assumption of normality and were log transformed prior to statistical tests. The statistical analyses of the MEG data were focused on examining the frequency, timing and amplitude of beta suppression and rebound within the data. Here, we first identified for each participant the peak frequency of the beta suppression and rebound by averaging the TFR data in the time-window of −0.5–0.75 and 0.75–2 s, respectively. For the beta suppression, we determined the frequency showing the smallest value and then identified the peak suppression time by determining the time instances showing the lowest power level at the peak frequency for each individual. The peak amplitude of beta suppression was then determined as the power value at the peak frequency and timing. For the beta rebound, the process was equivalent, but here we first identified the frequency showing the highest power value in the rebound time interval for each individual and then determined the peak rebound time as the time instance showing the highest value at this frequency.

For the SARCOPENIA study, Mann-Whitney *U* tests were used to determine potential between-group differences. For the PASSWORD study, repeated measures Analysis of Variance tests (1 group * 3 time) were used to determine potential differences over time. Mauchly's test evaluated sphericity and Greenhouse-Geisser degrees of freedom corrections were used when a statistically significant deviation was observed. Where a statistically significant F-value was observed, Least Significant Differences post hoc tests were used to determine the source of the difference. This post hoc test was chosen to prevent Type II error, given the small sample size. Effect sizes for between-group and post hoc (over-time) comparisons were calculated according to Hedge's *g*, where small effects <0.2, medium effects 0.2–0.8, and large effects >0.8 are classified. Data are presented as mean and SD as non-transformed values. Statistical significance was set to *P* < 0.05.

## 3 Results

### 3.1 Isometric plantarflexion performance

In SARCOPENIA, long-term resistance-trained individuals showed lower CV in relaxation time for both 15% (U = 66.0, *P* = 0.021, *g* = 0.159) and 50% (U = 65.0, *P* = 0.027, *g* = 0.119) of MVC (Table 1) compared to age-matched controls. All other comparisons were equivalent (Table 1).

**Table 1:** Plantarflexor force data (mean ± SD) from the SARCOPENIA and PASSWORD studies. The task was to exert force to a target level (15% and 50% of MVC) as fast as possible and then stop contracting.

		Baseline			*P*-value¤
**SARCOPENIA**					
Max Force (N)	Trained	1001.6 ± 350.3			0.237
	Untrained	822.7 ± 223.4			
Average Force 50% (N)	Trained	548.0 ± 197.8			0.083
	Untrained	430.9 ± 124.3			
CV Force 50%	Trained	0.098 ± 0.030			0.203
	Untrained	0.116 ± 0.035			
Error Ratio 50%	Trained	1.091 ± 0.071			0.274
	Untrained	1.050 ± 0.087			
Average RFD 50% (N·s^−1^)	Trained	2624.2 ± 1170.0			0.122
	Untrained	1814.9 ± 518.9			
CV RFD 50%	Trained	0.184 ± 0.057			0.762
	Untrained	0.204 ± 0.056			
Relaxation time 50% (ms)	Trained	348.6 ± 119.2			0.360
	Untrained	513.5 ± 273.8			
CV Relaxation time 50%	Trained	0.220 ± 0.103			0.027
	Untrained	0.391 ± 0.197			
Contraction time 50% (ms)	Trained	629.5 ± 135.6			0.360
	Untrained	784.7 ± 287.3			
Average Force 15% (N)	Trained	203.9 ± 123.3			0.274
	Untrained	153.4 ± 62.3			
CV Force 15%	Trained	0.173 ± 0.064			0.408
	Untrained	0.195 ± 0.048			
Error Ratio 15%	Trained	1.318 ± 0.536			0.829
	Untrained	1.207 ± 0.187			
Average RFD 15% (N·s^−1^)	Trained	1151.8 ± 1047.8			0.897
	Untrained	884.2 ± 455.0			
CV RFD 15%	Trained	0.333 ± 0.149			0.829
	Untrained	0.331 ± 0.078			
Relaxation time 15% (ms)	Trained	326.6 ± 119.2			0.360
	Untrained	343.0 ± 108.2			
CV Relaxation time 15%	Trained	0.239 ± 0.106			0.021
	Untrained	0.381 ± 0.123			
Contraction time 15% (ms)	Trained	556.8 ± 164.0			0.897
	Untrained	534.6 ± 116.5			

		**Pre-training**	**Mid-training**	**Post-training**	**P-value¤**
**PASSWORD**					
Max Force (N)		747.4 ± 217.4	792.5 ± 246.0	799.7 ± 215.5	0.250
Average Force 50% (N)		399.6 ± 128.0	386.7 ± 134.2	407.4 ± 119.4	0.500
CV Force 50%		0.120 ± 0.047	0.125 ± 0.042	0.122 ± 0.079	0.753
Error Ratio 50%		1.066 ± 0.099	0.999 ± 0.216	1.014 ± 0.061	0.287
Average RFD 50% (N·s^−1^)		1462.0 ± 766.3	1757.0 ± 868.1*	1889.0 ± 789.6*	0.003
CV RFD 50%		0.257 ± 0.088	0.202 ± 0.078	0.186 ± 0.086	0.001
Relaxation time 50% (ms)		487.9 ± 221.0	421.4 ± 157.8	495.3 ± 290.2	0.337
CV Relaxation time 50%		0.329 ± 0.140	0.334 ± 0.198	0.248 ± 0.150	0.073
Contraction time 50% (ms)		840.8 ± 251.2	714.8 ± 181.7	762.9 ± 310.8	0.035
Average Force 15% (N)		129.4 ± 48.8	127.9 ± 39.5	131.9 ± 43.2	0.881
CV Force 15%		0.229 ± 0.051	0.226 ± 0.038	0.198 ± 0.044	0.032
Error Ratio 15%		1.148 ± 0.210	1.107 ± 0.276	1.094 ± 0.155	0.592
Average RFD 15% (N·s^−1^)		682.6 ± 347.7	707.3 ± 303.3	773.5 ± 297.5	0.056
CV RFD 15%		0.301 ± 0.105	0.282 ± 0.061	0.251 ± 0.066	0.073
Relaxation time 15% (ms)		355.9 ± 188.2	334.3 ± 122.3	368.3 ± 240.4	0.924
CV Relaxation time 15%		0.337 ± 0.225	0.397 ± 0.259	0.316 ± 0.187	0.427
Contraction time 15% (ms)		579.5 ± 214.3	548.7 ± 144.6	571.2 ± 257.1	0.726

P-value for SARCOPENIA denotes between-group difference from Mann-Whitney U tests, while denoting a main effect for Time from repeated measures Analysis of Variance tests in PASSWORD. Max = maximum, RFD = rate of force development, CV = coefficient of variation. * = significant difference compared to Pre-training (P < 0.05).

Over the training period, PASSWORD participants improved their average RFD to 50% of MVC (F_1.53, 29.15_ = 8.369, *P* = 0.003) from both Pre- to Mid- (*P* = 0.044, Δ% = 27 ± 41%, *g* = 0.462) and Pre- to Post-training (*P* < 0.001, Δ% = 35 ± 36%, *g* = 0.991). Also, the CV of average RFD to 50% of MVC reduced (F_2, 38_ = 8.000, *P* = 0.001) from both Pre- to Mid- (*P* = 0.013, Δ% = −15 ± 41%, *g* = 0.590) and Pre- to Post-training (*P* = 0.003, Δ% = −24 ± 33%, *g* = 0.743). Although a similar pattern was observed for contractions to 15% of MVC, these did not reach statistical significance (Table 1). Contracting to 15% of MVC did show a reduction in CV of average force (F = 3.769, *P* = 0.032), with significant reductions from Mid- to Post-training (P = 0.013, Δ% = −11 ± 19%, *g* = 0.591). Contraction time for 50% of MVC trials shortened (F_2, 38_ = 3.678, *P* = 0.035) from Pre- to Mid-training (*P* = 0.018, Δ% = −11 ± 22%, *g* = 0.555), but contraction time for 15% of MVC trials did not (Table 1).

### 3.2 Beta-band oscillations during and after isometric contraction

**Fig. 2:**
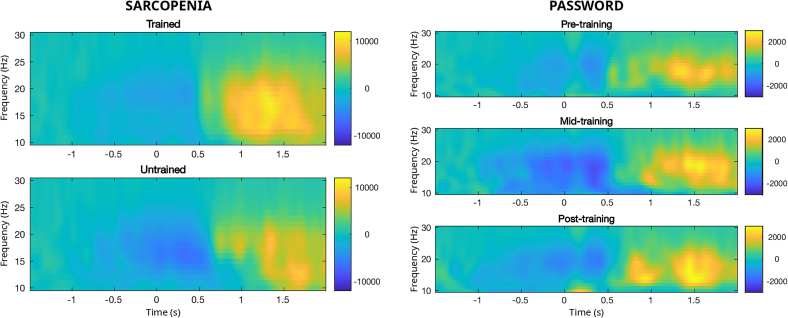
Grand average Time-Frequency Representations from 10 to 30 Hz (1 Hz intervals) for SARCOPENIA (left) and PASSWORD (right) data sets. First, the peak rebound frequency was identified for each individual, and then peak rebound time was identified between 750 and 2000 ms after contraction initiation for this frequency. The colour scaling indicates the magnitude of the Time-Frequency Representations s (fT^2^/cm^2^).

Due to large motion-induced artifacts during the 50% of MVC trials interfering with signals at the time and frequencies of interest, only the 15% of MVC trials were assessed from the MEG data. For beta oscillations, potential differences in peak frequency, amplitude and time for both suppression and rebound were evaluated (Fig. 2).

Peak rebound frequency did not change (F_2, 38_ = 0.171, *P* = 0.844) throughout PASSWORD (Pre-training: 19.1 ± 5.5 Hz, Mid-training: 18.5 ± 4.6 Hz, Post-training: 18.4 ± 5.7 Hz). Similarly, there were no differences between groups in SARCOPENIA in peak rebound frequency (trained: 17.2 ± 4.3 Hz, untrained: 16.8 ± 4.4 Hz, *P* = 0.829, *g* = 0.088). Further, peak suppression frequency did not differ for any comparison in both data sets.

Peak suppression amplitude (trained: −5715 ± 3552 fT^2^/cm^2^, untrained: −8154 ± 8160 fT^2^/cm^2^, *P* = 0.762, *g* = 0.386) and rebound amplitude (trained: 20810 ± 25,206 fT^2^/cm^2^, untrained: 16210 ± 10,792 fT^2^/cm^2^, P = 0.762, *g* = 0.217) were equivalent between groups in SARCOPENIA. However, beta rebound showed a significant increase (F_2, 38_ = 4.867, P = 0.013) from Pre- to Post-training (Pre: 4639 ± 7271 fT^2^/cm^2^, Post: 7179 ± 12,986 fT^2^/cm^2^, P = 0.003, *g* = 1.152) in PASSWORD. No changes were observed for peak suppression amplitude in PASSWORD.

**Fig. 3:**
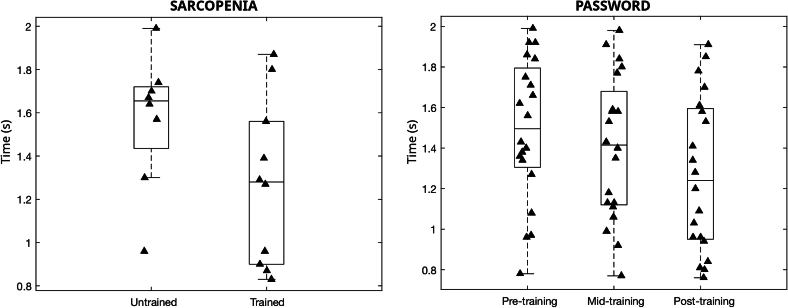
Peak rebound time (s) for SARCOPENIA (left) and PASSWORD (right) data sets. The box plots show the 25%, 50% and 75% quartiles of the data with the whiskers representing the minimum and maximum individual values falling within 1.5 times the interquartile range along with individual participant data.

Peak rebound time did not differ between-groups in SARCOPENIA (trained: 1.27 ± 0.38 s, untrained: 1.57 ± 0.31 s, *P* = 0.083, *g* = 0.814), but there were reductions observed in PASSWORD (F_2, 38_ = 3.342, *P* = 0.046) from Pre- to Post-training (Pre: 1.49 ± 0.35 s, Post: 1.27 ± 0.38 s, *P* = 0.039, *g* = 0.477) (Fig. 3). No differences in peak suppression time were observed for either data set.

## 4 Discussion

The present study examined beta-band oscillations during voluntary isometric plantarflexion contractions in the resistance-trained and untrained state by leveraging two experimental models: a between-group comparison of 10-year resistance trained versus untrained older adults and a within-group comparison before and after a 12-month multicomponent intervention. Both short- and long-term resistance-training led to lower variability in force rise and relaxation as well as contraction time during a fast, targeted plantarflexion task. There was also some evidence for improved RFD from short-term training. However, the cross-sectional comparison of long-term resistance-trained versus untrained individuals did not show the same pattern. Therefore, our first hypothesis on faster RFD and more accurate force production was only partly confirmed. Similarly, in relation to our second hypothesis, peak rebound amplitude was greater following short-term resistance training but the between-group difference in the cross-sectional (SARCOPENIA) study was not significant. Further no differences nor changes were observed in beta suppression amplitude. Therefore, our hypotheses can be partly accepted. A consistent finding from the MEG data was the timing of the peak rebound, with an average latency difference of ∼0.23 s between the trained and untrained state, although the change was not statistically significant between long-term trained and untrained individuals (P = 0.083).

### 4.1 Fast, targeted force production and resistance training

There were only few differences in force production characteristics in SARCOPENIA, despite the long-term resistance-trained participants being stronger in gym-based exercises than the untrained controls (currently unpublished finding). Possible reasons for the limited group differences may have been insufficient statistical power, given that the significant differences observed for variability in relaxation time had small effect sizes (i.e. Hedge's *g* < 0.2). The relatively low participant numbers in SARCOPENIA are understandable given the unique experimental design consisting of an initial one-year supervised resistance training intervention and ten-year follow-up of 19 regularly resistance-trained individuals. Applying MEG inclusion and exclusion criteria further reduced this number to 10 suitable individuals compared to 8 untrained controls from the same study.

Short-term resistance training did lead to improvements in RFD, as has been substantially demonstrated in various muscle groups [32], [33], [34], [35], [36]. RFD, particularly over durations <100 ms [37], are thought to be primarily regulated by neural factors such as motor unit discharge rate [38], [39]. However, whether modulation of motor unit behaviour is produced by supraspinal or spinal level adaptation is currently unresolved.

In relation to this evidence of modulated motor unit behaviour, the clearest evidence of improvements in performance accompanying resistance training was observed in force variability. Hunter et al. [14] showed that declined force steadiness between young and older adults was likely influenced by motor unit discharge variability and the present study showed that resistance training in older adults reduced variability in both force rise and relaxation suggesting improved sensorimotor control (i.e. afferent-efferent interaction).

### 4.2 Plantarflexion movement-related beta-band oscillations

Beta suppression is suspected to relate to preparation for movement [40] and reduced beta power is accompanied by greater corticospinal excitability [41]. The equivalent amplitude and timing of beta suppression in the trained and untrained state may indicate a similar ability to overcome beta power-related ongoing GABA_A_ activity [8] and initiate efferent drive. Aging has been shown to accompany higher pre-movement beta power [3], [4], [9], [10], and resistance training may not have been sufficient to counteract this potential factor in movement-related beta suppression amplitude.

On the other hand, beta rebound amplitude has been shown to distinguish between healthy individuals and patient groups [11], [12], with increased rebound accompanying rehabilitation success [13]. Since beta-band modulation occurs during passive and various afferent stimulation conditions (e.g. [2], [3]), this may relate to accuracy/appropriateness of the movement response. Force steadiness [11] and hand dexterity [12] were also shown to accompany greater beta rebound amplitude suggesting its importance in motor control. In the present study, we observed increases in beta rebound amplitude from short-term resistance training that occurred in parallel with improved force variability. It is worth noting that both trained and untrained groups in SARCOPENIA had a higher rebound amplitude than the experimental group in PASSWORD (not statistically tested), which may indicate generally high functioning in this sample along with the limited differences in fast contraction performance. Potentially, our results indicate that resistance training is efficacious in synchronizing beta-band activity post-movement if there is already reduced, e.g. afferent, synchrony but it likely provides no further advantage over already well-functioning older individuals.

The clearest effect on beta-band oscillations was observed in the timing of the peak rebound. Resistance training experience led to a faster peak latency. This may have been expected given higher RFD [6], [42] and shorter contraction/relaxation times [40]. Nevertheless, functional relevance of shorter rebound latency has been shown in a reaching accuracy task [43], and in the present study it may be one key factor in the reduced force and force timing variability in the trained state. It is important to note that the data in the present study does not allow determination of the directionality of behavioural and brain data, i.e. is the faster peak rebound because the contraction occurs and ends (more systematically) earlier or does the contraction occur/end earlier because sensorimotor functioning is more efficient. Notwithstanding, it would be important for future studies to explore the connection between afferent functioning and post-movement beta rebound timing in more detail, perhaps from single trials, to understand its role in motor control. In animal models, prolonged bursting post-movement accounted for longer latency beta rebound [44]. Such prolonged rebound may affect subsequent motor actions [45], where fast, repetitive actions are needed as in fall prevention. Currently, it may be speculated that reductions in peak beta rebound latency accompanying resistance training imply faster conduction velocity or, more likely, afferent processing prior to or within the cortex. This improvement may affect control over fast motor actions and potentially increased ability to perform sequential actions.

Aging has also been shown to reduce beta frequency during voluntary actions [4]. While the present study did not compare young and older adults, the results show no changes in the peak frequency of either suppression or rebound due to resistance training. To our knowledge, the functional relevance of beta frequency has not been resolved, and the improved fast, targeted ankle plantarflexion performance occurred in absence of changes suggesting limited importance here. Where measurement sensitivity of beta frequency may be questionable [46], the measure has been shown to be reliable in test-retest settings [29] even after one year [31]. Therefore, it could be argued that the present studies would have identified changes in beta frequency should this have occurred.

### 4.3 Study strengths and limitations

The present study has clear strengths that are worth highlighting. First, the SARCOPENIA study included a unique cohort of 10-year resistance-trained older adults, whose systematic training was tracked. Second, combining this data with a short-term supervised resistance- and walking-training intervention for one year provides evidence of replication and merging patterns of adaptation. Third, the present study uses a novel MEG-compatible force dynamometer that enables isometric contractions to be produced in the magnetically shielded room without disturbance to cortical signals. As a result of this first study, we show that low force levels (here 15% of maximum) can be produced in a plantarflexion action successfully without signal artifacts or critical head movements, but artifacts occurred during the higher force (50% of maximum) task. Subsequent studies may wish to test different actions, such as ankle dorsiflexion or knee extension/flexion, and/or various force levels.

Nevertheless, the present study contains limitations that should be discussed. First, magnetic resonance images would have been necessary for accurate source location analyses of suppression and rebound. This information could have provided valuable insight into the precise location of suppression and rebound adaptation (e.g. sensorimotor cortex, pre-motor cortex, M1) as well as oscillation timing. Second, interpreting the present study contains uncertainty whether the observed changes in movement-related beta-band oscillations are task-specific or a result of general changes in brain processing. We would have needed to assess resting state data to get a better handle on potential generic changes to brain processing. It may be speculated that the relatively short-term duration of PASSWORD could limit global changes in brain processing from occurring, which perhaps reduces this limitation's likelihood. However, a lack of control group reduces confidence in such suggestions as this also limits the certainty in attributing the observed changes to the intervention. Finally, the plantarflexors are a difficult muscle group to isolate as they are active during posture and locomotion. Postural control and walking are reliant on afferent-efferent coordination, at least at the spinal level. Therefore, individuals in the untrained state are not completely ‘untrained’, and it is unsure whether daily afferent activity from locomotion would influence cortical adaptation. Additionally, most resistance training programs do not specifically target plantarflexors but concentrate on hip and knee dominating exercises (e.g. squat, leg press, knee extension/flexion). This may have reduced the potential differences between long-term trained versus untrained individuals. Here, the short-term resistance training intervention has perhaps targeted plantarflexion more specifically as it included walking along with a calf-raise exercise.

## 5 Conclusion

Our data provide significant new insight into sensorimotor processing of movement and the potential impact of physical activity (either through sustained or lack of exercise) rather than age per se. Data on time to peak beta rebound suggests slowing of neuronal processing in the untrained state, but this latency can be ‘rescued’ by physical training. Given the lack of difference between-groups in SARCOPENIA and limited improvements in plantarflexion force production in PASSWORD, it is not clear whether resistance training (focussed primarily on proximal muscles) aiming to increase force production capacity drove the observed modifications in beta-band oscillations or whether higher physical activity levels in general are sufficient to maintain/improve sensorimotor cortical functioning. Nevertheless, both short- and long-term effects suggest faster and more synchronous sensorimotor processing in response to voluntary movement as well as lower force variability accompanying resistance training in older age.

## CRediT authorship contribution statement

**S. Walker:** Writing – review & editing, Writing – original draft, Visualization, Methodology, Funding acquisition, Formal analysis, Data curation, Conceptualization. **S. Sipilä:** Writing – review & editing, Methodology, Funding acquisition, Conceptualization. **H. Pesonen:** Writing – review & editing, Methodology, Data curation. **I.M. Tarkka:** Writing – review & editing, Methodology, Data curation. **J.P. Ahtiainen:** Writing – review & editing, Methodology, Conceptualization. **T. Parviainen:** Writing – review & editing, Methodology, Funding acquisition, Conceptualization. **J. Kujala:** Writing – review & editing, Writing – original draft, Visualization, Methodology, Funding acquisition, Formal analysis, Conceptualization.

## Funding

This work was supported by grants to SW (#350528) and SS (#296843) and by profiling funding on “Brain changes across the life-span” awarded to the 10.13039/501100005222University of Jyväskylä (#311877) from 10.13039/501100004787the Research Council of Finland.

## Declaration of competing interest

The authors declare that they have no known competing financial interests or personal relationships that could have appeared to influence the work reported in this paper.
